# Elevation of the *TP53* isoform *Δ133p53β* in glioblastomas: an alternative to mutant p53 in promoting tumor development

**DOI:** 10.1002/path.5111

**Published:** 2018-07-31

**Authors:** Marina Kazantseva, Ramona A Eiholzer, Sunali Mehta, Ahmad Taha, Sara Bowie, Imogen Roth, Jean Zhou, Sebastien M Joruiz, Janice A Royds, Noelyn A Hung, Tania L Slatter, Antony W Braithwaite

**Affiliations:** ^1^ Department of Pathology, Dunedin School of Medicine University of Otago Dunedin New Zealand; ^2^ Maurice Wilkins Centre for Molecular Biodiscovery New Zealand; ^3^ Department of Neurosurgery Southern District Heath Board New Zealand; ^4^ Department of Radiology Southern District Health Board New Zealand; ^5^ Jacqui Wood Cancer Centre, Division of Cancer Research University of Dundee UK

**Keywords:** glioblastoma, *TP53* isoform, *Δ133p53β*, mutant *TP53*, tumor‐associated macrophages, CSF1R, PDL1

## Abstract

As tumor protein 53 (p53) isoforms have tumor‐promoting, migration, and inflammatory properties, this study investigated whether p53 isoforms contributed to glioblastoma progression. The expression levels of full‐length *TP53α* (*TAp53α*) and six *TP53* isoforms were quantitated by RT‐qPCR in 89 glioblastomas and correlated with *TP53* mutation status, tumor‐associated macrophage content, and various immune cell markers. Elevated levels of *Δ133p53β* mRNA characterised glioblastomas with increased CD163‐positive macrophages and wild‐type *TP53*. *In situ*‐based analyses found *Δ133p53β* expression localised to malignant cells in areas with increased hypoxia, and in cells with the monocyte chemoattractant protein *C‐C motif chemokine ligand 2* (*CCL2*) expressed. Tumors with increased *Δ133p53β* had increased numbers of cells positive for macrophage colony‐stimulating factor 1 receptor (CSF1R) and programmed death ligand 1 (PDL1). In addition, cells expressing a murine ‘mimic’ of Δ133p53 (Δ122p53) were resistant to temozolomide treatment and oxidative stress. Our findings suggest that elevated *Δ133p53β* is an alternative pathway to *TP53* mutation in glioblastoma that aids tumor progression by promoting an immunosuppressive and chemoresistant environment. Adding *Δ133p53β* to a *TP53* signature along with *TP53* mutation status will better predict treatment resistance in glioblastoma. © 2018 The Authors. *The Journal of Pathology* published by John Wiley & Sons Ltd on behalf of Pathological Society of Great Britain and Ireland.

## Introduction

Glioblastoma is the most lethal glial tumor in adults, in part because it lacks effective treatment 1. Underpinning a better outcome from this disease is a better understanding of how individual tumors will progress and respond to treatment.

Tumor protein 53 is a strong tumor suppressor and without it, cancer is highly likely 2. Mice lacking *Trp53* develop T‐cell lymphoma, and humans with inherited *TP53* mutations develop multiple cancers including glioblastoma 3, 4. Despite an increasing list of functions attributed to wild‐type p53, identifying *TP53* mutants alone has limited power in predicting patient outcomes 5, 6. One reason proposed is that other *TP53* signatures, such as p53 isoforms, are present which may disrupt or alter p53 function 5.

At least 12 isoforms of *TP53* exist including the Δ133p53 family, which are missing the first 132 amino acids of p53 and are further distinguished by alternative splicing at the *C‐*terminus resulting in three isoforms, Δ133p53α, Δ133p53β, and Δ133p53γ 5, 7. Pro‐tumorigenic functions have been attributed to Δ133p53 isoforms including cell cycle progression 8, 9, 10, anti‐apoptotic activity 11, angiogenesis 12, invasive and migratory functions 13, 14, inflammation 9, increased DNA repair 15, and decreased chemosensitivity 16.

Mice continuously expressing elevated levels of a ‘mimic’ of Δ133p53 (Δ122p53) have a profound pro‐inflammatory phenotype characterised by increased serum cytokines such as CCL2 and interleukin 6 (IL‐6) 9, 13. The pro‐inflammatory function of Δ133p53 isoforms may contribute to cancer progression, as Δ122p53 mice have an early onset of tumors compared with p53‐null mice, and the tumors have inceased angiogenesis and a greater propensity to metastasise 9, 13. Moreover, the metastasis is largely dependent on IL‐6 17.

Δ133p53 function is enhanced when wild‐type p53 is present, as is evident in gastric cancer inflammation in response to *Helicobacter pylori* infection 18, and in scratch‐wound assays 13. If *Δ133p53* levels were increased on a wild‐type *TP53* background in cancer, an analysis of this isoform along with that for *TP53* mutations may improve the ability of *TP53* to predict treatment responses.

Glioblastoma is an aggressive tumor, with approximately 20% of tumors containing *TP53* mutations 19. The majority of *TP53* mutations occur in a subset of tumors that use the alternative lengthening of telomeres (ALT) as an alternative telomere maintenance mechanism to telomerase activity (77% versus 19.4% of *TP53* mutations, respectively) 20. Recently, we reported different patient outcomes associated with subtypes of glioblastoma based on the telomerase maintenance mechanism and CD163 macrophage content of the tumor 21, 22. The three major subgroups highlight the heterogeneity of treating glioblastoma. Tumors that are ALT‐positive have the best patient outcome, but the median patient survival has not improved with the introduction of temozolomide 21, 23. Thus, ALT‐positive tumors may be less aggressive, but alternative treatments are required before improvements to the median survival will be made.

Mutant *TP53* is less frequent in the remaining two tumor subtypes. The second subtype (TEL) comprising telomerase‐positive tumors with a low content of macrophages has an improved median survival, with approximately 30% of patients showing longer‐term survival since temozolomide use 21, 22. Patients with a third subgroup (TELM) comprising telomerase activity‐like tumors with a high content of macrophages (> 35% of the tumor) have not shown improvement with temozolomide treatment, and are now associated with the poorest outcome. New treatments are required for those with TELM tumors.

Here, we investigated whether *TP53* isoforms were increased in tumors with a high content of macrophages that resist current treatments (TELM). We report that the *Δ133p53β* isoform is associated with an immunosuppression and chemoresistance signature in TELM tumors.

## Materials and methods

### Patients and tissue specimens

Glioblastoma tissue samples were obtained from 89 individuals subtyped for the three major telomere maintenance mechanisms and macrophage content‐based subtypes 21, 22. Ethical approval (reference LRS/10/09/037/AM05 and MEC/08/02/061/AM01) was obtained in New Zealand and all procedures followed institutional guidelines. All individuals provided written informed consent.

### Preparation of RNA, cDNA synthesis, and real‐time qPCR analyses

Normal brain RNA was obtained from Ambion (Austin, TX, USA) and Clontech (Palo Alto, CA, USA). Total cellular and tissue RNA was prepared by a PureLink™ RNA Mini Kit (Invitrogen, Carlsbad, CA, USA) and 1 μg of total RNA was reverse‐transcribed using the qScript cDNA synthesis system (Quanta Biosciences, Gaithersburg, MD, USA).

Real‐time qPCR was performed with a LightCycler® 480 System (RochemDiagnostics, Basel, Switzerland) using SYBR Green Master Mix (TaKaRa Bio, Otsu, Japan). Reactions used 50 ng of cDNA, were run in duplicate, and a mean value of the two samples was calculated. Relative expression levels of each gene were quantified by the 2^−ΔCt^ method using *glyceraldehyde 3‐phosphate dehydrogenase* (*GAPDH*) as an endogenous control. The primers used for *GAPDH* were 5'‐GAAGGTGAAGGTCGGAGTC‐3' and 5'‐GAAGATGGTGATGGGATTTC‐3', and for *CDKN1A* they were 5'‐CTAATGGCGGGCTGCATCCA‐3' and 5'‐AGTGGTGTCTCGGTGACAAAGTC‐3', and *TP53* variants as previously described 24. A published nested PCR approach was used to confirm the presence of the *Δ133p53β* transcript in 20 glioblastomas 5.

### Immunohistochemical examination

For immunohistochemical (IHC) staining, 4‐μm sections from paraffin‐embedded tissues were used. The KJC8 antibody towards p53β was generously provided by the Bourdon Laboratory (University of Dundee, Ninewells Hospital and Medical School, Dundee, UK) and was detected using EnVision Dual Link (Dako, Glostrup, Denmark) and diaminobenzidine chromogen (DAB), with DAB enhancer (Leica Biosystems, Wetzlar, Germany). The CA9 antibody (MRQ‐54; Cell Marque, Rocklin, CA, USA) was used to detect CA9‐positive cells using an automated IHC method (BOND RX automated stainer; Leica Biosystems, Wetzlar, Germany).

### RNAscope *in situ* hybridisation

A custom probe to the unique region of *Δ133p53* and *p53β* was made by Advanced Cell Diagnostics for use in the RNAscope assay (Advanced Cell Diagnostics, Newark, CA, USA). The probe was designed against *Δ133p53β* reference sequence DQ186651.1, with the probes requested to be made between the following nucleotides: +97 to +277 (including the sequence unique to *Δ133p53* isoforms and designed to exclude the upstream *AluJb* repeat). To increase the amount of sequence available to design the probes against, nucleotides between +847 and + 1001 (region in *p53β* isoforms) were also included. Other probes included were to human and murine *CCL2*, to human and murine *ubiquitin C* (*UBC*) as a positive control for RNA quality, and to the bacterial gene *DapB* as a negative control.

Formalin‐fixed, paraffin‐embedded cell line clots, and brain tumors were cut into 5‐μm sections. The RNAscope method used the manual assay 2.5 protocol with Protease Plus reagent for protein digestion and the 2.5HD Brown reagent kit for detection of the probe according to the manufacturer's instructions. The assay for brain tumors was subjected to a modification to the RNAscope protocol suggested for brain tissue and involved a reduced protease digestion time (25 min instead of 30 min). Following the addition of DAB, DAB enhancer was added as above.

### Slide analyses

Positive cells were identified using the Aperio Scanscope CS digital pathology system (Aperio, Vista, CA, USA). Two examiners evaluated slides in a blinded fashion. The percentage of positive cells was identified using the Aperio RNA ISH Algorithm (for the RNAscope slides) and the Aperio Nuclear Algorithm (for the KJC8‐stained slides). The median percentage of positive cells from five fields at ×400 magnification was calculated.

### Sequencing *TP53* mutations

DNA was extracted from frozen tumors. To detect *TP53* mutations, PCR‐amplified exons 4–9 of *TP53* were sequenced using previously published primers 25. Purified PCR products were subjected to Sanger sequencing.

### Drug treatment

Temozolomide and *tert*‐butyl hydroperoxide (Sigma‐Aldrich, St Louis, MO, USA) were added to the mouse p53‐null fibroblast cell line 10.1 (from Professor Wolfgang Deppert, Heinrich‐Pette‐Institut, Hamburg, Germany) that was transduced with either a retrovirus expressing *Δ122p53* or a control vector 13. At different time‐points over 48 h following drug treatment, viable cells were counted using trypan blue exclusion. 10.1 cells were maintained in Dulbecco's modified Eagle's medium (Gibco, Waltham, MA, USA) supplemented with 10% fetal calf serum at 5% CO_2_ and 37 °C. The concentration of temozolomide chosen was the minimal dose of a range found in the cerebrospinal fluid of patients with glioblastoma undergoing temozolomide treatment and was that used to investigate temozolomide resistance in brain tumor‐initiating cells 26, 27.

### Statistical analyses

Continuous data were compared using one‐way ANOVA for multiple comparisons or *t*‐tests. The comparison based on the *TP53* mutant status of tumors was made using an unpaired *t*‐test with Welch's correction. Categorical data were compared using chi‐squared tests.

A complete linkage hierarchical clustering was performed using the hclust() function in R after ranking the mRNA expression of *TAp53*, *Δ40p53*, *Δ133p53*, *p53α*, and *p53β*; per cent CD163 immune cell content; and telomere maintenance mechanism subtype in ascending order using the rank() in R. Patient survival analyses were performed using the homoscedastic Student's *t‐*test. Statistical analyses were performed using GraphPad Prism software version 7 (Graphpad Inc, La Jolla, CA, USA) and R 28 statistical software. *p* < 0.05 was taken as statistically significant.

## Results

### Elevated *Δ133p53β* mRNA levels in glioblastomas with a high macrophage content

**Figure 1:**
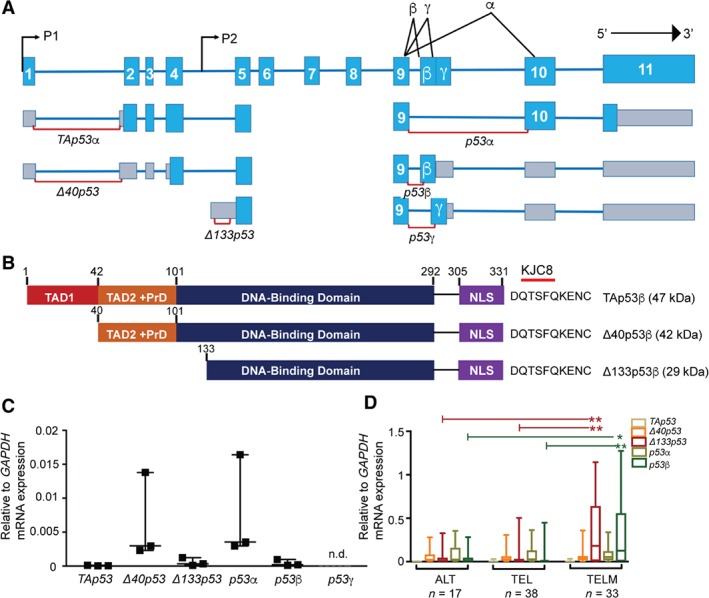
Δ*133p53*β is increased in glioblastoma with a high macrophage content. (A) Schematic diagram of the human *TP53* gene structure. The *TP53* gene can encode for at least nine transcripts (*TAp53α*, *TAp53β*, *TAp53γ*, *Δ40p53α*, *Δ40p53β*, *Δ40p53γ, Δ133p53α*, *Δ133p53β*, and *Δ133p53γ*) generated by alternative splicing (α, β, and γ) and alternative promoter usage (P1 and P2). Six RT‐qPCR reactions were used in this study; three detected the transcripts encoding the *N*‐terminus of the corresponding p53 isoform and three detected the *C*‐terminus of the corresponding *TP53* isoform (shown as red lines). The light blue region represents the coding exons and the grey regions represent the untranslated regions. (B) Region recognised by the rabbit polyclonal antibody KCJ8, designed to specifically detect the β isoforms of p53. (C) Scatter plots show *TP53* (*TAp53*, *Δ40p53*, *Δ133p53*, *p53α*, and *p53β*) variant expression relative to *glyceraldehyde‐3 phosphate dehydrogenase* (*GAPDH*) in normal brain tissue (*n* = 3). Symbols show individual samples; horizontal lines represent median values and vertical lines represent the range. (D) Box and whiskers plots show *TP53* (*TAp53*, *Δ40p53, Δ133p53*, *p53α*, and *p53β*) variant expression in three subgroups of glioblastomas (*n* = 89), with a high and a low macrophage content, determined by RT‐qPCR analysis. ALT, alternative lengthening of telomere tumors with a low content of tumor‐associated macrophages; TEL, telomerase‐positive tumors with a low content of tumor‐associated macrophages; TELM, telomerase‐positive tumors with a high content of tumor‐associated macrophages.

To determine if increased *TP53* isoform expression was associated with immune infiltration in glioblastoma, 89 tumors representative of the ALT, TEL, and TELM subgroups and three normal brain samples were analysed by quantitative PCR assays. The *TP53* gene can express at least nine transcript variants encoding at least 12 protein isoforms. Thus, to distinguish and quantitate the different *N*‐terminal and *C*‐terminal transcripts subclasses, we used six RT‐qPCR assays designed to detect either full‐length *TP53* (*TAp53*), *Δ40p53*, *Δ133p53*, *p53α*, *p53β*, or *p53γ*, respectively (Figure 1A) 24. Other tools used to detect p53 isoforms included the KJC8 antibody, which detects all β‐containing isoforms (Figure 1B).

Normal brain had low *Δ133p53* mRNA and higher *Δ40p53* mRNA (Figure 1C). Of the three 3′ *TP53* transcripts, *p53α* was highest, *p53β* was minimal, and *p53γ* was not detected (Figure 1C). For the tumor samples, the TELM tumor subset had significantly higher expression of *Δ133p53* and *p53β* compared with ALT (*p =* 0.002 and *p =* 0.031, respectively) and TEL tumors (*p =* 0.004 and *p =* 0.009, respectively; Figure 1D).

The increased *Δ133p53* and *p53β* suggest that *Δ133p53β* mRNA is the predominant isoform in TELM tumors. To support this, there was a significant and positive correlation between *Δ133p53β* and *p53β* expression in TELM tumors (ρ = 0.96; *p* < 0.0001; supplementary material, Figure S1A, B). To confirm the presence of the *Δ133p53β* transcript, 20 tumors were assayed using nested PCR as previously described 5. A band corresponding to the size expected from the *Δ133p53β* transcript was present in 15 tumors with high *Δ133p53* expression and absent in five tumors with no *Δ133p53* and *p53β* (supplementary material, Figure S1C). *GAPDH* was selected as a reference gene as *GAPDH* levels had minimal variability in expression between normal and tumor tissues and between tumor subgroups with the majority of Ct values between 20 and 22 cycles (supplementary material, Figure S1D, E).

**Figure 2:**
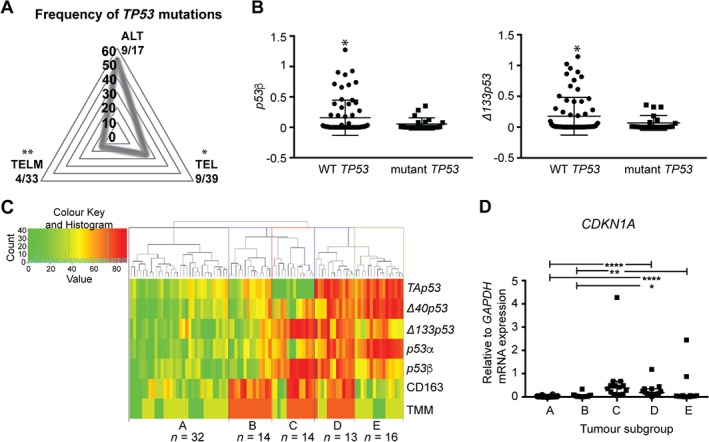
Δ*133p53*β is increased in glioblastoma with wild‐type *TP53*. (A) Radar plot depicting the frequency of *TP53* mutations in ALT, TEL, and TELM glioblastoma subtypes. (B) *p53β* (left) and *Δ133p53* (right) expression comparison between tumors with wild‐type *TP53* and those with *TP53* mutations. (C) Heat map and rank hierarchical clustering of the 89 tumors clustered by mRNA expression of *TAp53*, *Δ40p53*, *Δ133p53*, *p53α*, *p53β*, immune marker CD163, and the telomere maintenance mechanism (TMM) subtype showed five distinct clusters (named groups A–E). TMM: dark green = ALT‐positive tumor; light green = TEL‐positive tumor; orange = TELM‐positive tumor. (D) Expression of a well‐known p53‐induced gene, *cyclin dependent kinase inhibitor 1A*, (*CDKN1A*) amongst the five subgroups identified from the hierarchical clustering. *p < 0.05, **p < 0.01, ***p < 0.001, ****p < 0.0001.

Increased *Δ133p53* promoter activity occurs *in vitro* with wild‐type *TP53* 7. To determine if this is the case in glioblastoma, *TP53* mutations (missense, frame shift, or non‐sense) were identified in the cohort and compared with *Δ133p53β* expression. TELM tumors had the lowest *TP53* mutation frequency (Figure 2A). *p53β* expression, to represent *Δ133p53β*, was compared between tumors with wild‐type and those with mutant *TP53*. Increased *p53β* and *Δ133p53* occurred in tumors with wild‐type *TP53* and was minimal in tumors with mutant *TP53* [*p =* 0.015 (*p53β*) and *p =* 0.018 (*Δ133p53*); Figure 2B].

An unsupervised rank ordered hierarchical clustering was performed as a separate method of subtyping glioblastoma to distinguish the characteristics of tumors with increased *Δ133p53β*, CD163 immune cell content, and the telomere maintenance mechanism. This clustering analysis generated five tumor groups (Figure 2C) designated by similarity as group A (*n* = 32; 36%), group B (*n* = 14; 16%), group C (*n* = 14; 16%), group D (*n* = 13; 15%), and group E (*n* = 16; 18%).

The subgroups (C and D) with high *Δ133p53* and *p53β* had a high content of tumor‐associated macrophages. In group C, 64% of the tumors were TELM and in group D, 77% of the tumors were TELM tumors. Group D also showed high expression of the other *TP53* transcripts including high *TAp53, Δ40p53*, and *p53*α. Group E was characterised by high expression of *TAp53, Δ40p53*, and *p53*α, and low expression of *Δ133p53* and *p53β*. Group A had low expression of all the *TP53* transcripts.

Groups C and D had functional p53, as indicated by increased expression of a key downstream target of p53, *cyclin‐dependent kinase inhibitor 1A* (*CDKN1A*, Figure 2D). This is consistent with these tumors, with high *Δ133p53* and *p53β* and a high macrophage content, having a wild‐type *TP53* gene.

Individuals with group C tumors had increased survival with temozolomide treatment (either concurrent or adjuvant or both) compared with individuals who received no temozolomide (supplementary material, Figure S2). No treatment‐related response was evident for individuals with group A, B, D, and E tumors.

We conclude that elevated levels of *Δ133p53β* mRNA are found in two subgroups of glioblastomas (groups C and D) with a high content of infiltrating immune cells and wild‐type p53 function.

### Elevated *Δ133p53β* mRNA occurs in malignant cells

**Figure 3:**
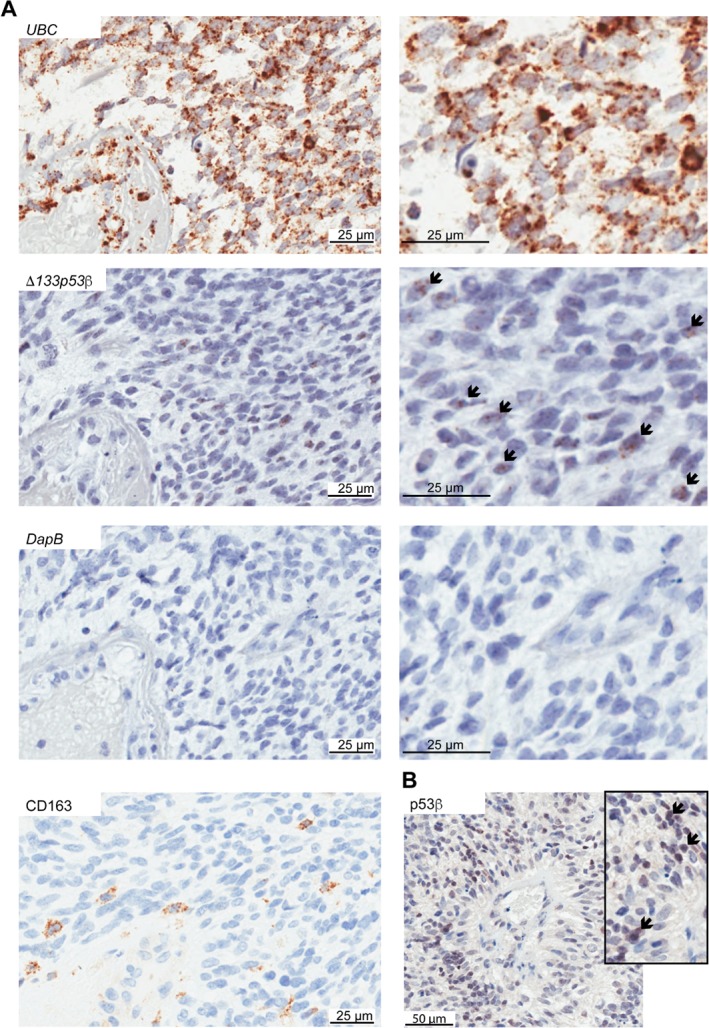
Δ133p53β is located in malignant cells in glioblastoma. (A) RNAscope detection of *Δ133p53β* in FFPE brain tumor tissues. Chromogenic staining (DAB) of brain tumor tissues hybridised with probes to *ubiquitin C* (*UBC*) as a positive control (top panel), probes against *Δ133p53β* (middle panel), and probes to the bacterial gene *DapB* as a negative control (bottom panel) are shown. Images taken at the same magnification but enlarged are shown on the right of the corresponding panel to better illustrate positive cells. To provide greater evidence that *Δ133p53β* was present in malignant cells, areas of the tumor were examined with minimal CD163‐positive cells (bottom left panel). Nuclei were counterstained with haematoxylin. Boxed areas show enlarged images to highlight *Δ133p53β* in tumor cells palisading from a necrotic blood vessel. (B) Immunohistochemistry using the antibody KJC8 to detect p53β in glioblastoma (left panel), and image enlarged (inset) to highlight positive cells in tumor cells palisading from a blood vessel. Arrows indicate positive cells.

To determine the cell types expressing *Δ133p53β* in glioblastoma, RNAscope and IHC using the KJC8 antibody to p53β isoforms were performed on 20 glioblastomas (five from groups A–D) 5. Due to the heterogeneity of glioblastoma and the difficultly of distinguishing all malignant cells in areas with a high content of macrophages without additional markers, regions of the tumors were selected with minimal CD163 infiltration (Figure 3A).

*Δ133p53β* mRNA was present in areas of pseudopalisading cells in all tumors from groups C and D (Figure 3A). The mean percentage of positive cells was 19% and 10%, respectively (supplementary material, Figure S3A). These cells also stained positive by IHC, where p53β was detected in cell nuclei (Figure 3B). Using the antibody towards p53β isoforms, the mean percentage of positive cells was 29% for tumors in group C and 18% for tumors in group D (supplementary material, Figure S3A).

Since pseudopalisading cells in glioblastoma are thought to comprise of migrating cancer cells 29, increased *Δ133p53β* mRNA is thus most likely expressed in cancer cells. In tumors from groups A and B, *Δ133p53β* was not evident in malignant cells from seven tumors. Four tumors, three from group B and one from group A, showed faint staining in malignant cells with the p53β antibody, or with RNAscope.

Other cell types were Δ133p53β‐positive by IHC. Ten tumors had regions of endothelial cells with positive cytoplasmic staining (supplementary material, Figure S3B). In 14 tumors (across all tumor groups), nuclear staining was present in some large cells that were neuronal specific enolase‐positive, CD163‐negative, and CD45‐negative, and thus likely to be neurons (supplementary material, Figure S3C).

The finding of *Δ133p53β* expression in malignant cells palisading around blood vessels suggested that *Δ133p53β* may be increased with hypoxia, as hypoxia is often associated with pseudopalisading necrosis 30, 31. To address this, tumors expressing *Δ133p53β* were stained for CA9 to highlight hypoxic areas of the tumor. *Δ133p53β* was prominent in CA9‐positive areas (supplementary material, Figure S4).

### *Δ133p53β* expression is associated with an immunosuppressive environment

**Figure 4:**
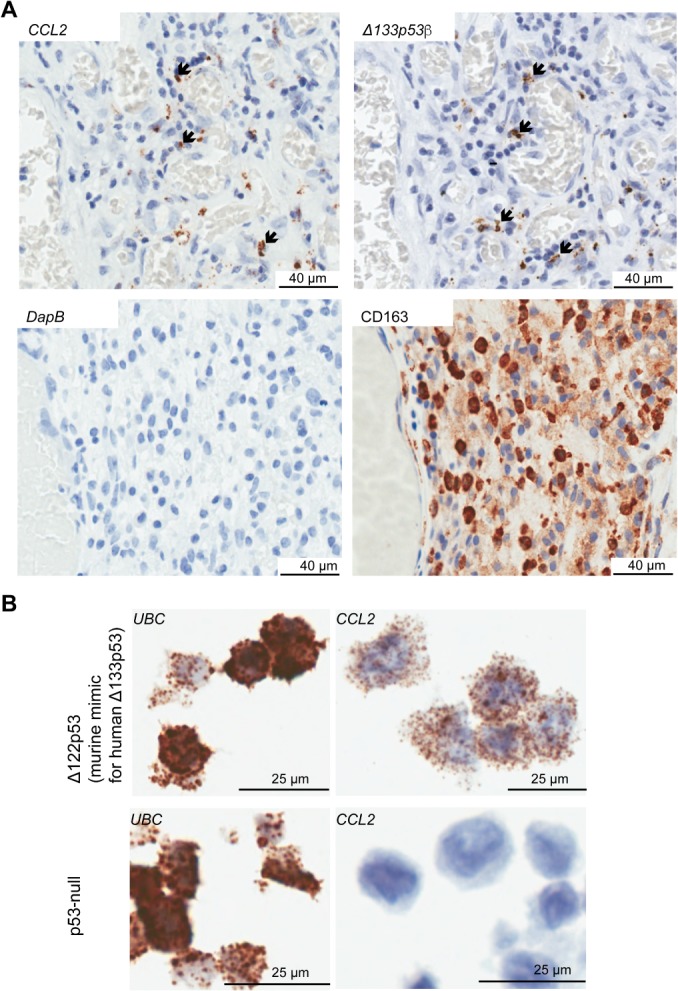
Δ*133p53*β correlates with a macrophage recruitment signature in glioblastoma. (A) In glioblastoma tissue sections, areas with CD163 macrophages identified using immunohistochemistry were correlated with monocyte chemoattractant protein *CCL2* expression and *Δ133p53β* using RNAscope. The absence of staining in the *DapB* control excluded non‐specific staining. (B) Mouse 10.1 cells engineered to express a ‘mimic’ of Δ133p53 (Δ122p53) had increased *CCL2* expression using RNAscope compared with cells expressing the vector alone (p53‐null). *Ubiquitin C* (*UBC*) was used as a positive control for the RNAscope assay.

A third non‐neoplastic cell type expressing *Δ133p53β* was evident from the RNAscope assay, where *Δ133p53β*‐positive stromal cells in areas around blood vessels associated with lymphocytes in tumors from groups C and D were identified (Figure 4A). This suggested that *Δ133p53β* may contribute to the expression of a key chemokine in macrophage recruitment 32, 33. To determine if increased *CCL2* was a consequence of Δ133p53β function, the mouse 10.1 p53‐null fibroblast cell line engineered to express the Δ133p53 ‘mimic’ (Δ122p53) was measured for *CCL2* by RNAscope 13. A marked increased in *CCL2* was present in cells expressing Δ122p53 and was absent in control cells expressing the control vector only (Figure 4B).

**Figure 5:**
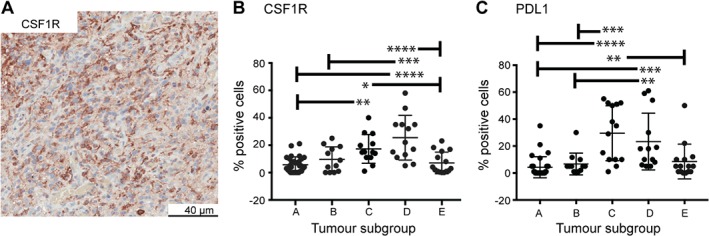
Δ*133p53*β correlates with an immunosuppressive signature in glioblastoma. (A) Immunohistochemistry for CSF1R (colony‐stimulating factor 1 receptor). (B) Comparison of the amount of CSF1R‐positive cells amongst the five glioblastoma subgroups identified using hierarchical clustering. (C) Comparison of the amount of PDL1 (programmed death ligand 1)‐positive cells amongst the five glioblastoma subgroups identified using hierarchical clustering. *p < 0.05, **p < 0.01, ***p < 0.001 ****p < 0.0001.

Considering that CCL2 and CD163 are characteristic of an immunosuppressive signature, additional markers were included to further characterise the extent of the immunosuppressive environment. Colony‐stimulating factor‐1 (CSF1) controls the differentiation, proliferation, and survival of macrophages by binding to CSF1R, expressed on macrophages and their progenitors 34. CSF1R was detected using IHC (Figure 5A). In groups A, B, and E, almost all the tumors showed a low percentage of CSF1R‐positive cells. In groups with increased *Δ133p53β* (C and D), individual tumors showed a range of CSFR1‐positive cells, but overall the percentage of CSF1R was increased in group D compared with groups A, B, and E (*p* < 0.001), and increased in group C compared with groups A and E (*p* < 0.05; Figure 5B).

Increased PDL1 in cancer activates the PD1–PDL1 checkpoint pathway leading to decreased cytokine production in T cells 35. PDL1 was detected using IHC (supplementary material, Figure S5). In groups A, B, and E, almost all the tumors showed a low percentage of PDL1‐positive cells. In groups C and D, individual tumors showed a range of PDL1‐positive cells, but overall the percentage of PDL1 was increased in group C compared with groups A, B, and E (*p* < 0.01) and increased in group D compared with groups A and B (*p* < 0.01 and Figure 5C).

In summary, glioblastoma subgroups with increased *Δ133p53β* mRNA were located in cells similar to those with *CCL2* expression and an increased immunosuppressive phenotype with increased CSF1R and PDL1‐positive cells. This evidence suggests that Δ133p53β contributes to tumor development by promoting an immunosuppressive environment.

### Δ122p53 confers a survival advantage upon temozolomide treatment and induction of oxidative stress

**Figure 6:**
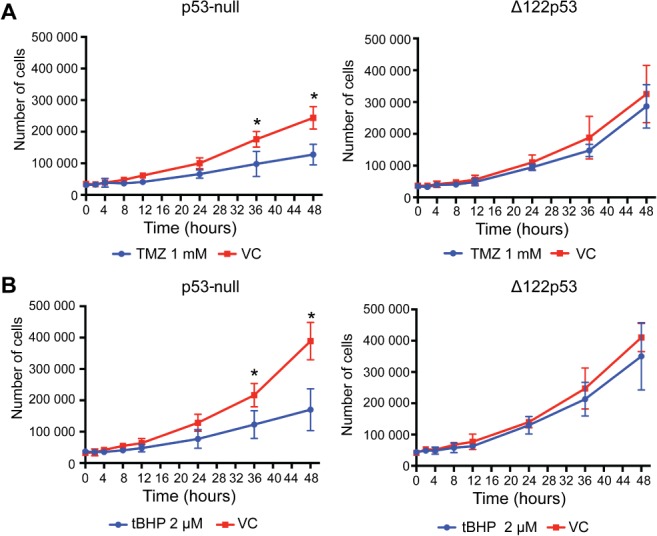
A mimic of *Δ133p53* function had reduced cytotoxicity to temozolomide and oxidative stress. Mouse 10.1 cells engineered to express a mimic of Δ133p53 (Δ122p53) and control cells expressing the vector (p53‐null) alone were treated with temozolomide (A) and *tert*‐butyl hydroperoxide to induce oxidative stress (B). The amount of viable cells was counted following trypan blue application. VC, vehicle control. Results represent the mean and standard deviation from three separate experiments.

To determine if Δ133p53β could contribute to the associated temozolomide resistance in TELM tumors (supplementary material, Figure S2), 10.1 cells expressing Δ122p53 were treated with temozolomide. Using 1 mm temozolomide, Δ122p53 cells were still viable over the entire time course, with no significant difference compared with vehicle control cells (Figure 6A). In comparison, the vector only‐containing cells showed a significant reduction in viability at 36 and 48 h post‐temozolomide treatment (*p =* 0.044 and *p =* 0.01, respectively, and Figure 6A).

Oxidative stress is increased in glioblastoma 36, 37. To determine if Δ133p53β could increase survival in response to oxidative stress, Δ122p53‐ expressing 10.1 cells and the vector control cells were treated with *tert‐*butyl hydroperoxide (tBHP) to induce oxidative stress over a 48‐h time course 38, 39. At a 2 μm dose of tBHP, Δ122p53‐expressing cells were viable, with no difference across all time points compared with the vehicle control (Figure 6B). In comparison, the vector only‐containing cells showed a significant reduction in viability at 36 and 48 h post‐treatment (*p =* 0.048 and *p =* 0.013, respectively, and Figure 6B).

These results suggest Δ133p53β could reduce the sensitivity to temozolomide and promote cell survival under oxidative stress.

## Discussion

We have demonstrated that the Δ133p53β isoform is increased on a wild‐type *TP53* background in glioblastoma. Increased Δ133p53β may aid glioblastoma progression by promoting an immunosuppressive environment and a tumor that is resistant to treatment. This evidence further supports an emerging concept that analysis of *TP53* isoform expression should be included along with mutant *TP53* status to better capture *TP53* signatures that contribute to clinical outcome 40, 41.

The elevated *Δ133p53β* mRNA was not due to mutations affecting mRNA stability. Instead, a wild‐type *TP53* background contributed to increased *Δ133p53β*. Wild‐type, and not mutant, p53 led to increased *Δ133p53* promoter activity in reporter assays 7, 8. The finding of increased *Δ133p53β* associated with wild‐type *TP53* and minimal expression with mutant *TP53* in the current study further suggests a role for wild‐type p53 in aiding tumor progression through increased levels of functional Δ133p53β.

Normal brain had minimal *Δ133p53β*; thus, a wild‐type *TP53* background alone does not account for increased *Δ133p53β* in glioblastoma. This suggests that the tumor ‘microenvironment’ may be responsible. Consistent with this, in glioblastoma, areas with hypoxia were positive for *Δ133p53β*. Upon hypoxia, p53 accumulates 42 and can increase to levels similar to those obtained with gamma irradiation 43. However, unlike gamma irradiation, the p53 stabilised with hypoxia was not followed by an increase in effector proteins such as p21 and bax 43. This suggests that canonical wild‐type p53, but not its downstream signalling pathways required for tumor suppression, is increased with hypoxia. Considering that Δ133p53β can inhibit TAp53α‐mediated pathways 5, 10, 44, a scenario could be envisaged in which hypoxia leads to the increased *TAp53α*, which in turn leads to increased Δ133p53β promoting tumor progression, including reduced downstream signaling of p53. Whether and how hypoxia leads to increased *Δ133p53β* mRNA is undetermined at this stage. Some cells in close proximity to hypoxic regions with minimal CA9 staining also showed increased *Δ133p53β* mRNA.

We found a strong association between *Δ133p53β* mRNA levels in glioblastoma with increased tumor‐associated macrophage content, which in previous studies was associated with poor clinical outcome 21, 22. This evidence suggests that Δ133p53β has an additional role by increasing the recruitment of CD163 macrophages. We provide additional evidence that Δ133p53β may be contributing to macrophage infiltration in glioblastoma by increased *CCL2* expression, a key cytokine involved in macrophage recruitment 32, 33. Increased expression of *CCL2* was observed in Δ122p53 and not p53‐null cells, and CCL2 inhibition prevented the migration of Δ122p53‐expressing cells 13. Our data thus suggest that elevated Δ133p53β in glioblastoma leads to increased *CCL2* transcription, which in turn promotes CD163 macrophage infiltration to create an immunosuppressive environment. This hypothesis is supported by a study where G261 mouse glioblastomas grown on a CCL2‐deficient background failed to recruit immunosuppressive myeloid cells, and the finding that CCL2 was associated with increased recruitment of T regulatory cells 45.

Considering that many cells produce CCL2 including tumor cells, non‐malignant astrocytes, endothelial cells, and macrophages and microglia in a brain tissue context 46, how much of the CCL2 in glioblastoma may be attributed to Δ133p53β, and whether this contribution is sufficient to affect tumor progression, remains to be determined. We were not able to provide direct evidence that malignant cells with *Δ133p53β* were those producing *CCL2*, due to the difficulty in distinguishing malignant cells in areas with a high content of macrophages. However, given that other studies have found malignant cells in glioblastomas with a high content of macrophages that produce *CCL2* 45, it is likely that some malignant cells produce *Δ133p53β* and *CCL2*.

An increased immunosuppressive phenotype with increased Δ133p53β was further evident with increased PDL1 and CSF1R cells. This suggests that immunotherapies may be more likely to work for *Δ133p53β* tumors. Whether Δ133p53β contributes directly to increased PDL1 and CSF1R is unknown. The increased CSF1R could be a consequence of the type of macrophages recruited. The Δ133p53 isoforms and the animal model ‘mimics’ are associated with increased NFκβ signalling followed by increased cytokine production; so it is likely that Δ133p53β contributes directly to the immunosuppression in glioblastoma by influencing the cytokines produced in the tumor environment 9, 13, 18, 47, 48.

Δ133p53β is likely to be functional in glioblastoma, as evidenced by the positive immunostaining using an antibody that detects all p53β isoforms. The increased Δ133p53β in TELM malignant cells is an important distinction from TEL tumors, which apart from the increased macrophage content are similar 21. The findings from this study suggest that Δ133p53β may reduce the sensitivity to temozolomide and further promote poorer patient survival by increasing treatment resistance. Including *Δ133p53β* mRNA quantitation along with *TP53* mutation status would provide a *TP53* signature that would predict resistance to treatment in both ALT and TELM tumors. The analysis using temozolomide was performed in a murine fibroblast cell line that mimicked Δ133p53β without wild‐type *Trp53* present. Considering that glioblastomas with increased *Δ133p53β* have wild‐type *TP53*, further analyses are required to determine if glioblastomas with elevated *Δ133p53β* are temozolomide‐resistant.

We cannot, with the tools available, exclude some contribution of the Δ160p53β isoform, which begins translation at an internal initiation codon 7, and although we describe tumors with increased *Δ133p53β* as having increased CD163 macrophages, we also cannot exclude the contribution from other CD163‐positive cells such as activated microglia 49.

Glioblastoma classification based on genome‐wide expression differences is more commonly used compared with that based on the telomere maintenance mechanism used here 50, 51, 52. How the *TP53* transcripts fit with the proneural, mesenchymal, classical, and neural gene expression‐based subtypes is yet to be determined. Considering that ALT‐positive tumors are likely to have *isocitrate dehydrogenase 1* mutations, as are proneural tumors, the proneural subtype is likely represented by groups A and E 53, 54.

In conclusion, our results suggest that elevated Δ133p53β occurs on a wild‐type *TP53* background and may contribute to multiple tumor‐promoting pathways in glioblastoma by contributing to the immunosuppressive and chemoresistant environment. Determining how Δ133p53 becomes established in cancer is key to understanding the disease progression. This study suggests a role for hypoxia.

## Author contributions statement

MK, RAE, SM, AT, SB, IR, JZ, NAH, and TLS participated in data generation. MK, RAE, SM, AT, SB, IR, NAH, JAR, TLS, and AWB participated in data analysis and interpretation. SMJ provided materials and technical support, and participated in critical review of the manuscript. TLS and AWB obtained the funding. MK, RAE, SM, TLS, and AWB participated in the concept and design of the study. MK, SM, RAE, TLS, and AWB wrote the manuscript. All authors critically evaluated the written manuscript.

**SUPPLEMENTARY MATERIAL ONLINE**

**Supplementary figure legends**

**Figure S1.** Confirmation of the *Δ133p53β* transcript in glioblastoma

**Figure S2.** Group C glioblastoma patients benefit from temozolomide treatment

**Figure S3.** Analysis of Δ133p53β in glioblastomas using RNAscope and IHC

**Figure S4.** Hypoxic areas in glioblastoma had increased *Δ133p53β*

**Figure S5.** Programmed death ligand 1 staining using immunohistochemistry in glioblastoma

## Supporting information


**Supplementary figure legends**

**Figure S1. Confirmation of the *Δ133p53β* transcript in glioblastoma. (A)** Pairwise Spearman correlation analysis for *p53β* expression relative to *Δ133p53* expression in TELM tumors. **(B)** The correlation matrix shows Spearman correlation coefficients for the pairing of *TP53* transcripts variables indicated on the *y*‐axis versus the *x*‐axis. Blue represents a positive correlation for a given gene pair and red represents a negative correlation. Asterisks indicate significant correlation at *p* of 0.01 (**), 0.001 (***), and 0.0001 (****). (**C)** A nested PCR approach was used to identify the *Δ133p53β* transcript in 20 glioblastomas. A band of approximately 748 bp was detected in 15 tumors with high expression and was absent in five tumors with no *Δ133p53* and *p53β* expression by quantitative PCR. M = molecular weight marker. *ACTB*, *actin beta*. (**D, E)** A single reference gene, g*lyceraldehyde 3‐phosphate dehydrogenase* (*GAPDH*), was used in this study for normalisation of *TP53* transcript data. *GAPDH* Ct values are shown for the tumor as a whole and compared with normal brain tissues (**D**), and in tumor subgroups (**E**) with the majority of Ct values between 20 and 22 cycles.

**Figure S2. Group C glioblastoma patients benefit from temozolomide treatment.** The *Y*‐axis shows the relative expression of *Δ133p53* and the *X*‐axis shows overall survival (months). Patients treated either alone or in combination concurrently or with adjuvant temozolomide are in blue and untreated patients are in red. The *X*‐axis cuts at the median *Δ133p53* expression = 0.0068. Homoscedastic Student's *t*‐test; *p* < 0.05 is considered significant. As only data for one untreated tumor are available in group D, statistical analyses were not performed on this group.

**Figure S3. Analysis of Δ133p53β in glioblastomas using RNAscope and IHC. (A)** The percentage of positive cells for *Δ133p53β* based on the RNAscope assay (left panel) and p53β based on immunohistochemistry (mean and standard deviation are shown for each tumor subgroup). (**B, C)** p53β staining in non‐malignant cells following immunohistochemistry using the KJC8 antibody. p53β in the cytoplasm of endothelial cells **(B)**. p53β in the nucleus of neurons **(C)**.

**Figure S4. Hypoxic areas in glioblastoma had increased *Δ133p53β*.** Hypoxic areas in glioblastoma tissue as indicated by positive carbonic anhydrase 9 (CA9) staining had *Δ133p53β* expression using RNAscope. Arrows indicate *Δ133p53β* positively stained cells.

**Figure S5. Programmed death ligand 1 staining using immunohistochemistry in glioblastoma. (A)** A tumor positive for programmed death ligand 1 **(**PDL1). **(B)** A tumor negative for PDL1.
