# Supplementary material for: Elevation of the TP53 isoform Δ133p53β in glioblastomas: an alternative to mutant p53 in promoting tumor development
Source: J Pathol. 2018 Jul 31;246(1):77–88. doi: 10.1002/path.5111 (PMC6120556; doi:10.1002/path.5111)
Supplement: Supplementary file 1 — Supplementary figure legends [file PATH-246-77-s002.doc]

**Supplementary figure legends**

**Figure S1. Confirmation of the *Δ133p53β* transcript in glioblastoma. (A)** Pairwise Spearman correlation analysis for *p53β* expression relative to *Δ133p53* expression in TELM tumours. **(B)** The correlation matrix shows Spearman correlation coefficients for the pairing of *TP53* transcripts variables indicated on the *y*-axis versus the *x*-axis. Blue represents a positive correlation for a given gene pair and red represents a negative correlation. Asterisks indicate significant correlation at *p* of 0.01 (**), 0.001 (***), and 0.0001 (****). (**C)** A nested PCR approach was used to identify the *Δ133p53β* transcript in 20 glioblastomas. A band of approximately 748 bp was detected in 15 tumours with high expression and was absent in five tumours with no *Δ133p53* and *p53β* expression by quantitative PCR. M = molecular weight marker. *ACTB*, *actin beta*. (**D, E)** A single reference gene, g*lyceraldehyde 3-phosphate dehydrogenase* (*GAPDH*), was used in this study for normalisation of *TP53* transcript data. *GAPDH* Ct values are shown for the tumour as a whole and compared with normal brain tissues (**D**), and in tumour subgroups (**E**) with the majority of Ct values between 20 and 22 cycles.

**Figure S2. Group C glioblastoma patients benefit from temozolomide treatment.** The *Y*-axis shows the relative expression of *Δ133p53* and the *X*-axis shows overall survival (months). Patients treated either alone or in combination concurrently or with adjuvant temozolomide are in blue and untreated patients are in red. The *X*-axis cuts at the median *Δ133p53* expression = 0.0068. Homoscedastic Student’s *t*-test; *p* < 0.05 is considered significant. As only data for one untreated tumour are available in group D, statistical analyses were not performed on this group.

**Figure S3. Analysis of Δ133p53β in glioblastomas using RNAscope and IHC. (A)** The percentage of positive cells for *Δ133p53β* based on the RNAscope assay (left panel) and p53β based on immunohistochemistry (mean and standard deviation are shown for each tumour subgroup). (**B, C)** p53β staining in non-malignant cells following immunohistochemistry using the KJC8 antibody. p53β in the cytoplasm of endothelial cells **(B)**. p53β in the nucleus of neurons **(C)**.

**Figure S4. Hypoxic areas in glioblastoma had increased *Δ133p53β*.** Hypoxic areas in glioblastoma tissue as indicated by positive carbonic anhydrase 9 (CA9) staining had *Δ133p53β* expression using RNAscope. Arrows indicate *Δ133p53β* positively stained cells.

**Figure S5.** **Programmed death ligand 1 staining using immunohistochemistry in glioblastoma. (A)** A tumour positive for programmed death ligand 1 **(**PDL1). **(B)** A tumour negative for PDL1.
