# Supplementary figures and images for: Elevation of the TP53 isoform Δ133p53β in glioblastomas: an alternative to mutant p53 in promoting tumor development
Source: J Pathol. 2018 Jul 31;246(1):77–88. doi: 10.1002/path.5111 (PMC6120556; doi:10.1002/path.5111)

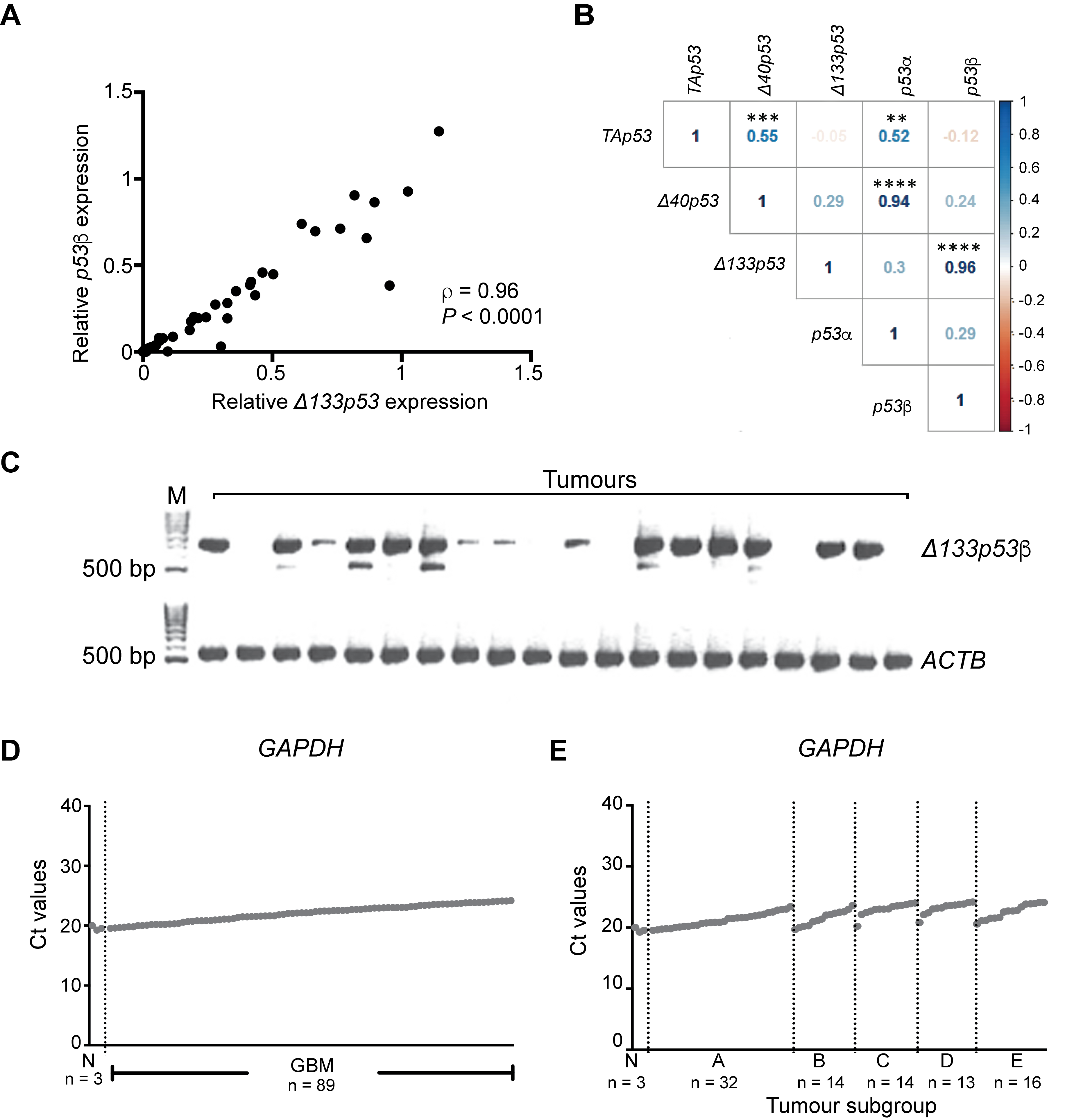

Supplement: Supplementary file 2 — Figure S1. Confirmation of the Δ133p53β transcript in glioblastoma. (A) Pairwise Spearman correlation analysis for p53β expression relative to Δ133p53 expression in TELM tumors. (B) The correlation matrix shows Spearman correlation coefficients for the pairing of TP53 transcripts variables indicated on the y‐axis versus the x‐axis. Blue represents a positive correlation for a given gene pair and red represents a negative correlation. Asterisks indicate significant correlation at p of 0.01 (**), 0.001 (***), and 0.0001 (****). (C) A nested PCR approach was used to identify the Δ133p53β transcript in 20 glioblastomas. A band of approximately 748 bp was detected in 15 tumors with high expression and was absent in five tumors with no Δ133p53 and p53β expression by quantitative PCR. M = molecular weight marker. ACTB, actin beta. (D, E) A single reference gene, glyceraldehyde 3‐phosphate dehydrogenase (GAPDH), was used in this study for normalisation of TP53 transcript data. GAPDH Ct values are shown for the tumor as a whole and compared with normal brain tissues (D), and in tumor subgroups (E) with the majority of Ct values between 20 and 22 cycles. [file PATH-246-77-s003.tif]

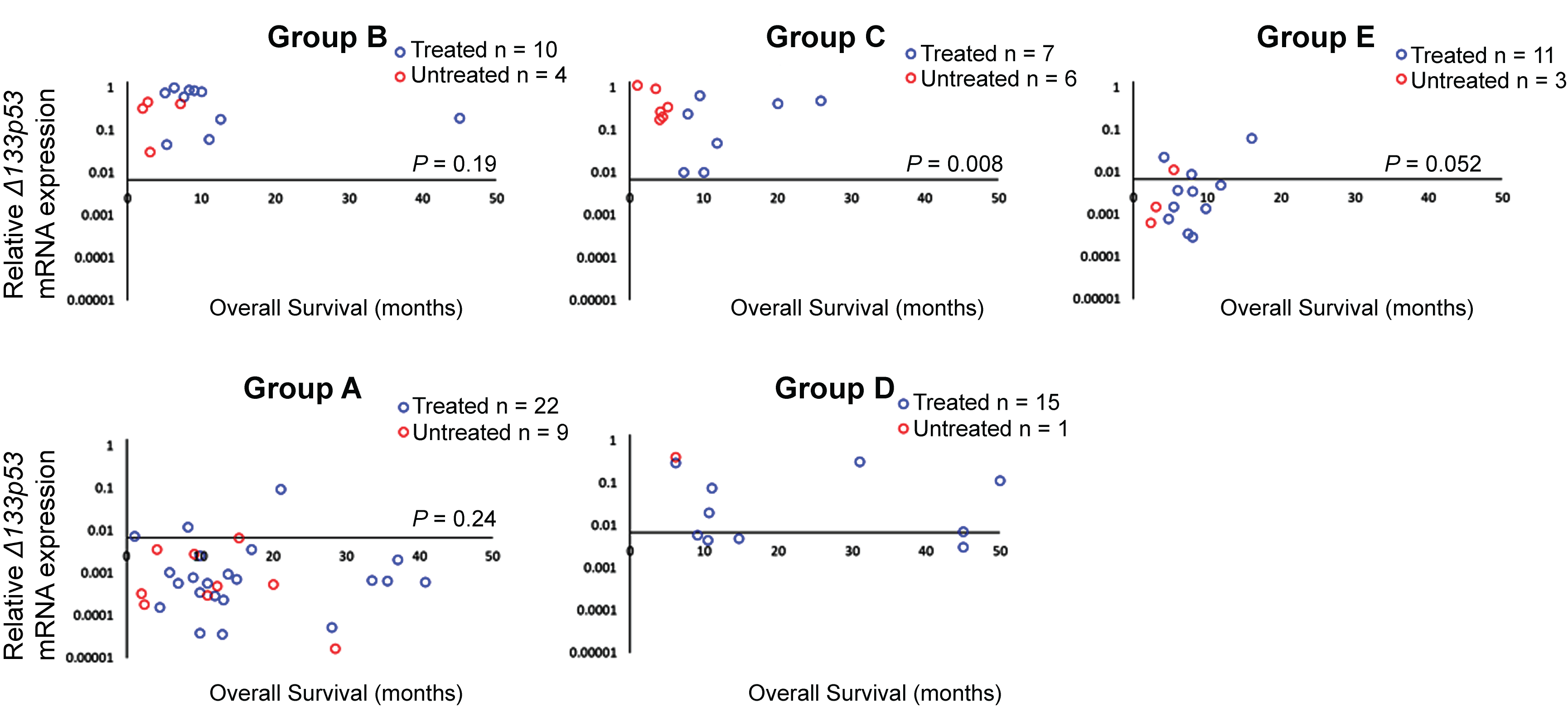

Supplement: Supplementary file 3 — Figure S2. Group C glioblastoma patients benefit from temozolomide treatment. The Y‐axis shows the relative expression of Δ133p53 and the X‐axis shows overall survival (months). Patients treated either alone or in combination concurrently or with adjuvant temozolomide are in blue and untreated patients are in red. The X‐axis cuts at the median Δ133p53 expression = 0.0068. Homoscedastic Student's t‐test; p < 0.05 is considered significant. As only data for one untreated tumor are available in group D, statistical analyses were not performed on this group. [file PATH-246-77-s004.tif]

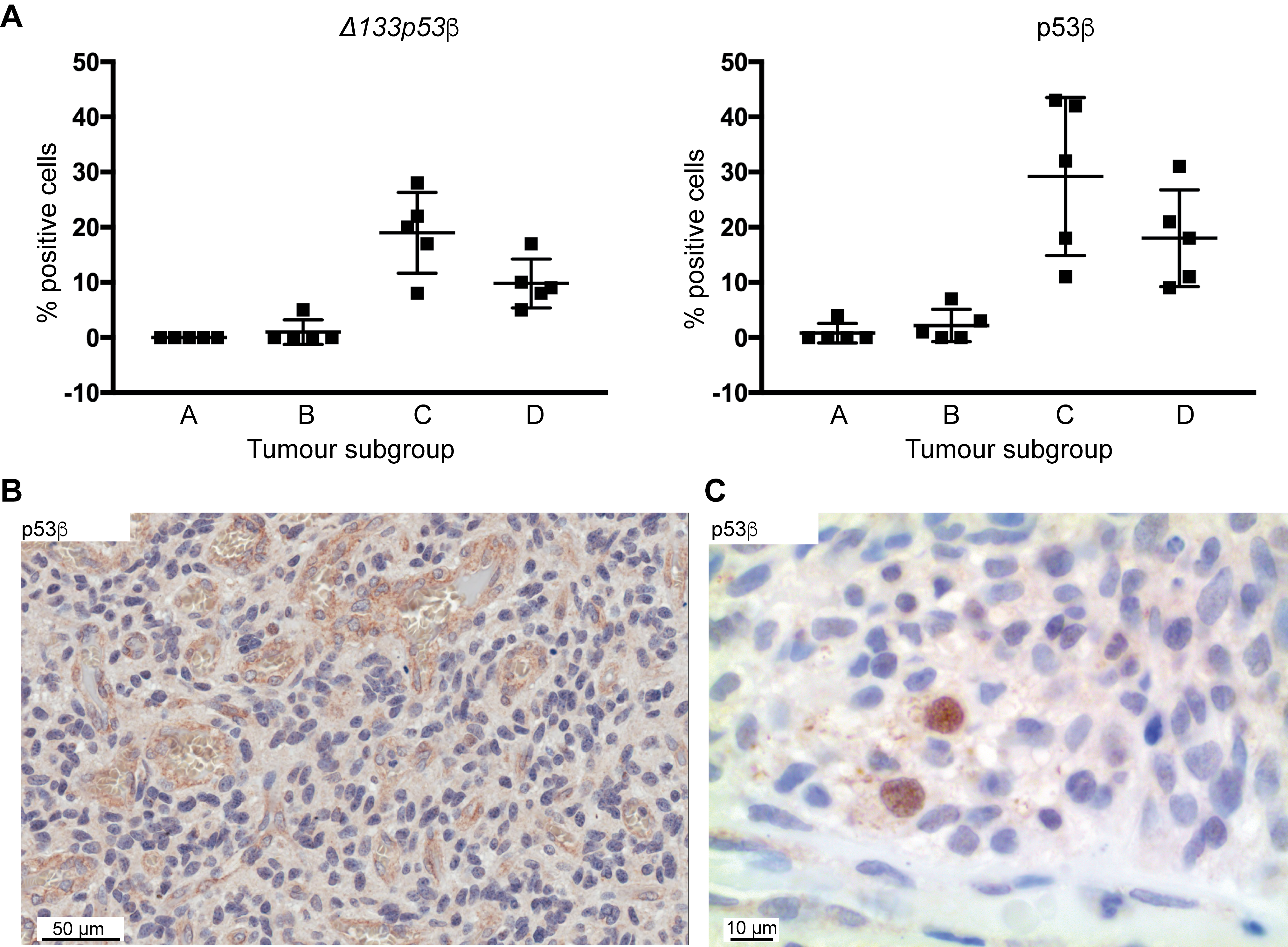

Supplement: Supplementary file 4 — Figure S3. Analysis of Δ133p53β in glioblastomas using RNAscope and IHC. (A) The percentage of positive cells for Δ133p53β based on the RNAscope assay (left panel) and p53β based on immunohistochemistry (mean and standard deviation are shown for each tumor subgroup). (B, C) p53β staining in non‐malignant cells following immunohistochemistry using the KJC8 antibody. p53β in the cytoplasm of endothelial cells (B). p53β in the nucleus of neurons (C). [file PATH-246-77-s005.tif]

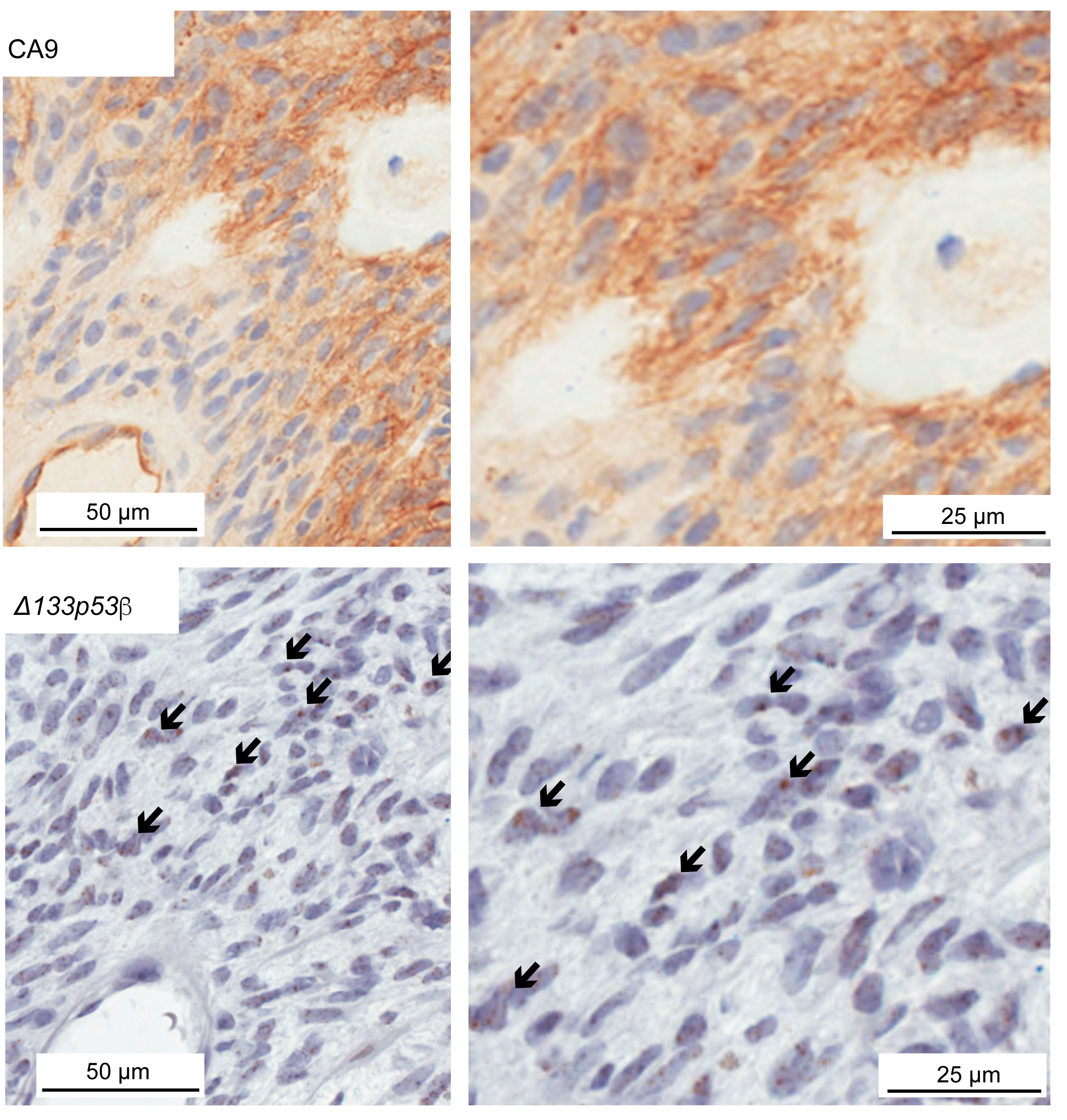

Supplement: Supplementary file 5 — Figure S4. Hypoxic areas in glioblastoma had increased Δ133p53β. Hypoxic areas in glioblastoma tissue as indicated by positive carbonic anhydrase 9 (CA9) staining had Δ133p53β expression using RNAscope. Arrows indicate Δ133p53β positively stained cells. [file PATH-246-77-s001.tif]

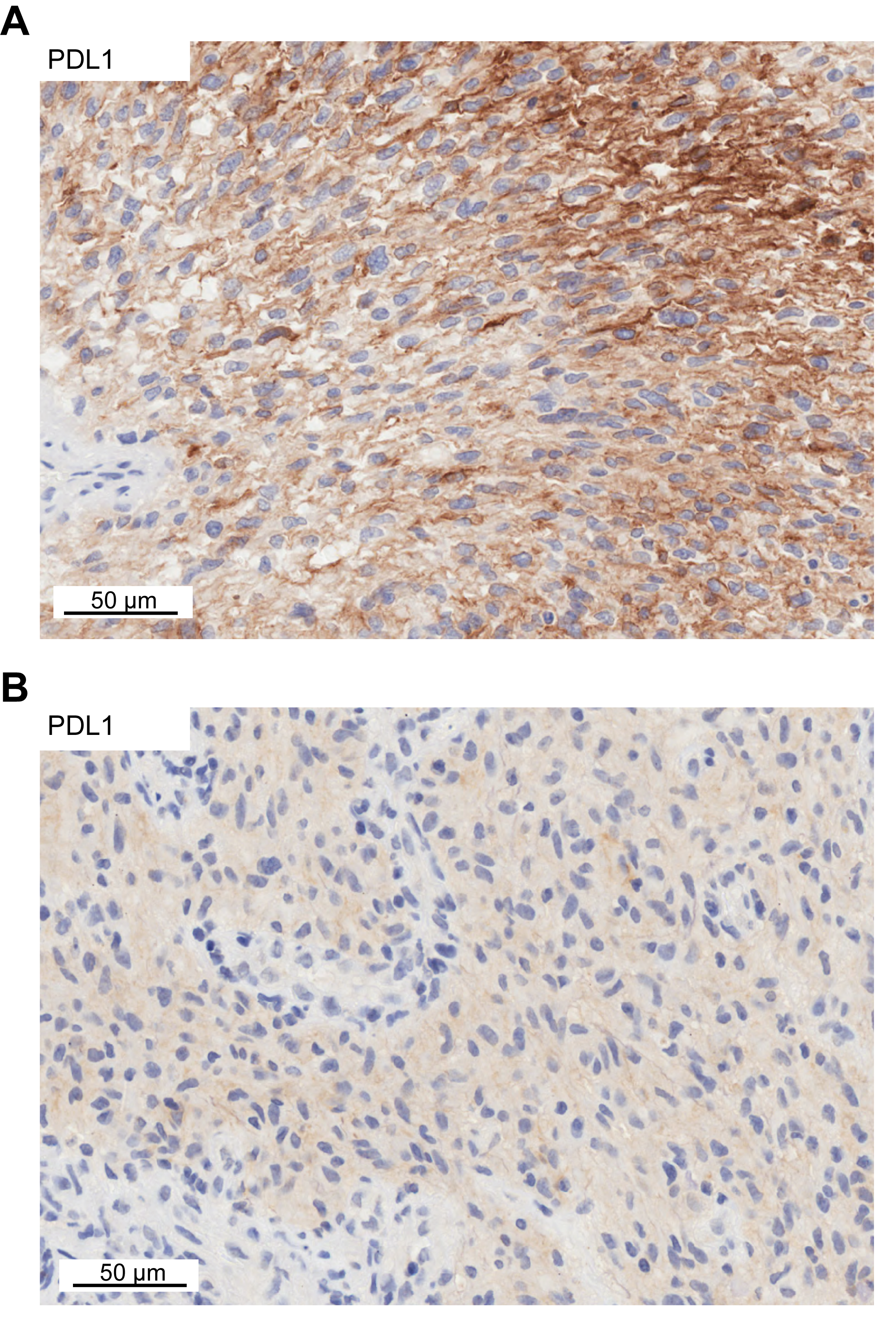

Supplement: Supplementary file 6 — Figure S5. Programmed death ligand 1 staining using immunohistochemistry in glioblastoma. (A) A tumor positive for programmed death ligand 1 (PDL1). (B) A tumor negative for PDL1. [file PATH-246-77-s006.tif]
